# Maturational Changes in Prefrontal and Amygdala Circuits in Adolescence: Implications for Understanding Fear Inhibition during a Vulnerable Period of Development

**DOI:** 10.3390/brainsci9030065

**Published:** 2019-03-18

**Authors:** Kelsey S. Zimmermann, Rick Richardson, Kathryn D. Baker

**Affiliations:** School of Psychology, University of New South Wales (UNSW), Sydney, NSW 2052, Australia; k.zimmermann@unsw.edu.au (K.S.Z.); r.richardson@unsw.edu.au (R.R.)

**Keywords:** fear extinction, adolescence, prefrontal cortex, amygdala

## Abstract

Anxiety disorders that develop in adolescence represent a significant burden and are particularly challenging to treat, due in no small part to the high occurrence of relapse in this age group following exposure therapy. This pattern of persistent fear is preserved across species; relative to those younger and older, adolescents consistently show poorer extinction, a key process underpinning exposure therapy. This suggests that the neural processes underlying fear extinction are temporarily but profoundly compromised during adolescence. The formation, retrieval, and modification of fear- and extinction-associated memories are regulated by a forebrain network consisting of the prefrontal cortex (PFC), the amygdala, and the hippocampus. These regions undergo robust maturational changes in early life, with unique alterations in structure and function occurring throughout adolescence. In this review, we focus primarily on two of these regions—the PFC and the amygdala—and discuss how changes in plasticity, synaptic transmission, inhibition/excitation, and connectivity (including modulation by hippocampal afferents to the PFC) may contribute to transient deficits in extinction retention. We end with a brief consideration of how exposure to stress during this adolescent window of vulnerability can permanently disrupt neurodevelopment, leading to lasting impairments in pathways of emotional regulation.

## 1. Introduction

Adolescence is a developmental period of “storm and stress,” characterised by a host of physical, cognitive, and emotional changes that permit a shift towards achieving independence while simultaneously opening a window of vulnerability to the damaging effects of external stressors [1,2,3]. Anxiety disorders that emerge during adolescence are a major concern, as they pose more societal burden and treatment cost than those emerging in adulthood [4]. One factor contributing to this burden is that, relative to adults, adolescents are far more prone to relapse following exposure therapy [5,6], the gold standard for treatment for anxiety disorders [7]. Identifying the neurological underpinnings that make adolescents particularly vulnerable to fear relapse will help to inform effective treatment approaches specifically tailored to the developing brain [8]. To this end, characterising the ways in which behaviour, learning, and memory are influenced by dynamic neurodevelopmental processes occurring during adolescence has become a topic attracting burgeoning international interest in recent years (e.g., a special issue devoted to Adolescence in *Neuroscience and Biobehavioral Reviews* in 2016 and a collection on Adolescence in *Nature* in 2018).

Extinction training is commonly used in an experimental setting to model the process of exposure therapy. Briefly, following Pavlovian conditioning, in which a neutral conditioned stimulus (CS; e.g., a white noise) is paired with an aversive unconditioned stimulus (US; e.g., a mild foot shock), an animal will exhibit fear responses to the CS alone. During extinction training, the CS–US contingency is degraded by repeatedly presenting the CS in the absence of the US. Eventually, CS-elicited fear is suppressed as the animal learns that it no longer predicts a threat. Some studies have shown that adolescents (both rodent and human) are delayed in reducing fear during extinction training, referred to as an impairment in within-session extinction [9,10]. Other studies have reported that even when within-session extinction is preserved, adolescents are far more prone to fear relapse than older or younger age groups when tested again at a later time point; that is, adolescents show deficits in extinction *retention* [11,12,13], reflective of the increased risk of relapse following exposure therapy. Discovering how the neural correlates underlying fear acquisition and extinction change over the course of development is a promising approach to understanding the cognitive and behavioural rigidity associated with aversive learning processes during adolescence.

The neurocircuitry underlying the acquisition and extinction of fear memories in adults has been extensively studied (and reviewed in detail in [14,15,16,17,18,19,20]). Two regions of particular interest are the prefrontal cortex (PFC), particularly the medial PFC (mPFC), and the amygdala. These highly interconnected forebrain structures regulate the formation and modification of associative memories, and their contributions to fear learning and extinction have been well established. Both structures undergo significant structural and functional changes over the course of development [21,22,23] that have the potential to fundamentally alter learning, memory, and behaviour. In this review, we summarise recent research describing developmental changes in PFC and amygdala regional plasticity, synaptic transmission, inhibition/excitation, and connectivity. We incorporate these findings into a structural framework modelling the ways in which these concomitant changes may underlie adolescent-specific deficits in extinction learning and retention, and discuss how behaviour can be impacted when the standard developmental trajectory is disrupted by exposure to external stressors.

## 2. Plasticity—Dendritic Spines

Developmental changes in neuroplasticity have been well-established; broad convention states that plasticity is highest in early life, when young animals need to quickly process large volumes of information about their environment, and decreases over development until reaching stable levels in adulthood [24]. However, different regions of the brain mature at different rates, meaning that later-developing regions like the PFC [25,26] are still highly plastic when other regions have largely stabilised. Understanding how neuroplasticity changes over development in the PFC and amygdala could help to identify regional imbalances in learning-dependent processes and reveal mechanisms underlying cognitive and behavioural rigidity in adolescence. 

One increasingly common approach used to characterise changes in neuroplasticity involves examining the density and stability of dendritic spines. Dendritic spines are the primary sites of glutamatergic synapses on excitatory principal neurons [27], like the pyramidal neurons of the mPFC and basolateral amygdala (BLA). These spines are highly dynamic, and their morphology, density, and stability (i.e., rate of turnover) change rapidly in response to plasticity-inducing forms of stimulation, including learning events. Although data concerning the relationship between learning and spine dynamics is thus far largely correlational (for review see [28]), increases in spine proliferation and reorganisation generally predict enhanced neuroplasticity, while spine elimination signals diminished capacity for change. 

### 2.1. Prefrontal Dendritic Spines

Postnatal development represents a particularly dynamic period of synapse and spine formation and elimination in the cortex across species [29,30]. Post-mortem analyses in humans show that following birth, spines and synapses on excitatory pyramidal neurons in the PFC massively proliferate until levels peak in mid-late childhood. Adolescence represents a period of dendritic pruning as neuronal processes are refined—during this period approximately half of all prefrontal spines and synapses are eliminated until adulthood, when levels stabilise and remain relatively constant [31,32]. This pattern of proliferation and pruning in the PFC is conserved across mammalian species, and has been demonstrated in the mPFC and dorsolateral PFC (dlPFC) of non-human primates [33,34,35], the mPFC (combined infralimbic [IL] and prelimbic [PL] subregions) of rats [23], and the PL and orbitofrontal cortex (OFC) of mice [36,37,38]. Importantly, when the PL and IL (rodent homologues of Brodmann Areas 32 and 25, respectively) are examined separately, it appears that the quadratic curve of spine density in the developing mouse mPFC is driven nearly entirely by the PL, with the IL showing little, if any, changes from early adolescence to adulthood [36]. Across regions, this means that in early adolescence, dendritic spine density in the PL is significantly higher than in the IL, whereas, in juveniles and adults, densities are comparable between regions. Given that the PL has been associated with fear expression, while the IL has been implicated in extinction [18], this temporary imbalance in plasticity and excitability between these two regions may help to explain the adolescent deficit in extinction retention, a hypothesis discussed in more detail in Section 2.3.

The transition from adolescence to adulthood involves circuit-specific changes in spine dynamics in the PFC which are concurrent with changes in afferent projections (discussed in detail in Section 5), suggesting there may be a relationship between spine fluctuations and the maturation of specific inputs to this region. This idea is supported by findings that transient increases in spine turnover in the PL of early adolescent mice occur in pyramidal neurons located in the same cortical layers that receive input from the ventral hippocampus and BLA (Layers II/III and V). Additionally, this adolescent increase in PL spine density and formation coincides with a peak in PL afferents arising from the ventral hippocampus and BLA [36]. It is likely that the observed reductions in spine plasticity and density in the PL between adolescence and adulthood are influenced by a combination of local changes in excitatory and inhibitory drive and circuit-specific reorganisation of connectivity. We discuss such changes and their implications for fear inhibition in adolescence in more detail in Section 4 and Section 5. 

### 2.2. BLA Dendritic Spines

Development of dendritic spines in the amygdala has been studied less extensively than in the PFC, but compelling data from rats suggest that the BLA shows a maturational pattern distinct from the proliferation-pruning trajectory of prefrontal areas. While PFC spines are dramatically eliminated during adolescence, spine density in the BLA shows a relatively linear increase from the juvenile period (childhood) to adulthood [23]. This study also reported slightly different patterns in BLA spine development between males and females; from adolescence to adulthood, there was a modest increase in density in males and an equally modest decrease in females. Concurrent with these changes in BLA spine density are fluctuations in amygdalar volume and the total number of cells within the amygdala. The volume of the lateral, basal, and central nuclei of the amygdala increases from Postnatal Day (P)7 to 35 in rats [39,40] (see Appendix A for a guide to postnatal development in rodents in postnatal days). Thereafter, amygdala volume decreases across adolescence to similar levels as in adulthood by P45 [39]. The decrease in amygdala volume across adolescence is likely due to small decreases in neuron number ([40] but see [39]) and reduced arborisation. It is interesting to note that volumetric analyses of amygdala development in humans appear to parallel the pattern of increasing amygdala volume from preadolescence to early adolescence reported in rats but not the later decreases across adolescence. Instead, the subtle differences between females and males in the trajectory of spine density changes across adolescence in rodents are reflected in the pattern of amygdala volume in human adolescents, although such studies in humans have often lacked the power to detect small sex differences. These studies show a linear increase in amygdala gray matter volume in boys between the ages of 4–18 that begins to slow around age 12, and a subtle quadratic curve for girls, with volume peaking at age 14 before slightly decreasing [41,42]. Results such as these highlight the need for increased research in the area of sex differences in neurodevelopment; this is an understudied topic that requires substantially more attention given the documented differences in prevalence of anxiety between males and females, with women and teenage girls having higher rates of anxiety than men and teenage boys, respectively [43,44,45,46]. In addition to more detailed investigations of sex differences in spine density and pruning in prefrontal-amygdala circuits across development (e.g., building on work in adults [47]), future analyses exploring whether there are differential trajectories in distinct populations of BLA neurons (e.g., “fear on” versus “fear off” neurons [48,49]) may provide additional insight into how BLA plasticity impacts fear and extinction across development.

### 2.3. Implications for Fear Learning and Inhibition

**Figure 1:**
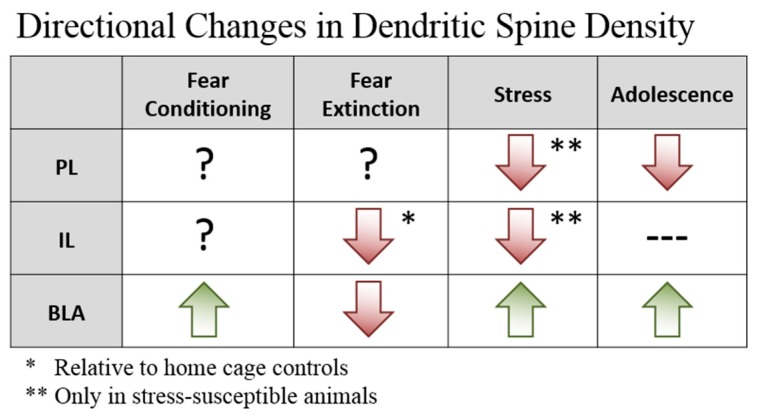
Summary of directionality of regional dendritic spine changes in response to fear conditioning, extinction, stress, and adolescence.

In terms of fear regulation, dendritic spines in the PFC and the BLA show evidence of remodelling following both fear conditioning and extinction in the adult brain [50,51]; however, the direction of the effect (formation vs. elimination of spines) is dependent on the region. In the frontal association cortex, fear conditioning induces spine elimination whereas extinction increases the rate of spine formation [50]. In contrast, fear conditioning is associated with increased spine density in the BLA; this effect is reversed if animals are given extinction training [51]. There may even be individual differences in BLA spine elimination after extinction that reflect the degree of within-session extinction by that animal, given reports that spine density in the BLA is positively correlated with fear expression during extinction, with increased spine density predicting higher levels of fear [52]. Interestingly, the correlation between fear expression and spine density in several brain regions appears to be mediated by stress exposure. Within the amygdala, stress exposure seems to recapitulate the effects of fear conditioning on dendritic spines, in that stressed animals (like fear-conditioned animals) show increased spine density in the BLA [53]. Stress also appears to have a direct impact on the interaction between fear expression and spine density in the mPFC. One study found that fear conditioning plus extinction was associated with decreased spine density in the IL relative to home cage controls [54]. However, when the animals were exposed to acute stress prior to extinction, a protocol that impaired both within-session extinction and extinction retrieval, fear expression during extinction retrieval was negatively correlated with IL spine density [54]. This suggests that increased IL spine density may be a mechanism of stress resilience. Notably, this correlation was not present in non-stressed animals. Although the effects of fear learning and extinction in the PL have not to our knowledge been explored, a similar interaction between dendritic spines and stress resilience is seen in this region; following chronic social defeat, stress-susceptible animals exhibited decreased PL spine density whereas stress-resilient animals showed no changes relative to non-stressed controls [55]. Taken together, it could be argued that spine hypertrophy in the BLA, and hypotrophy in the mPFC, is associated with states of negative emotional valence (i.e., fear and/or stress). As spine density is undergoing a period of growth and proliferation in the adolescent BLA and elimination in the PL (no major changes are detected in the IL), it is possible that the adolescent brain is similar to the brains of high-fear/stress-susceptible adults, rendering them more vulnerable to exaggerated spine loss in the mPFC and excessive hypertrophy in the BLA following adverse experiences. This may bias this age group towards a negatively-valenced state, making them more susceptible to pervasive, inflexible fear memories and more resistant to fear extinction. A novel question for future research is whether extinction in adolescent rats produces the same changes in spine density in the mPFC and BLA that are induced by extinction in adults, and whether such structural changes can be induced in adolescents by interventions that augment extinction retention. A summary of region-specific changes in dendritic spines in response to the conditions described above is provided in Figure 1. 

## 3. Plasticity—Learning-Dependent Changes in Neural Activity and Excitatory Transmission

Whereas changes in dendritic spine density and turnover in specific regions of the maturing brain can help to explain why adolescents may be biased towards negatively-valenced emotional learning, examination of immediate early genes and physiological indications of synaptic plasticity/long-term potentiation (LTP) in developing animals *around the time of a learning event* (e.g., fear extinction training) can help to identify discrete impairments in different components of memory acquisition, retrieval, and modification. Evidence that the adolescent brain shows altered synaptic plasticity after extinction has been clearly demonstrated by studies examining learning-induced immediate early gene induction and upregulation of protein markers implicated in neuroplasticity. Fear extinction in adults and pre-adolescents causes upregulation of learning-dependent markers of activity and plasticity, for example, c-Fos and phosphorylated mitogen-activated protein kinase (pMAPK) in the mPFC and BLA, that is not seen in adolescent animals [10,12,13]. Such findings indicate that adolescents may be less efficient at recruiting PFC-amygdala pathways during extinction, which may contribute to the extinction retention deficits seen in this age group. 

Experience-dependent plasticity in excitatory pyramidal neurons of the PFC and the BLA is widely considered critical for effective storage and expression of fear- and extinction-related memories [56,57] and appears to be dramatically disrupted in adolescence. In juvenile and adult animals, fear conditioning induces an increase in spontaneous excitatory postsynaptic currents (sEPSC) amplitude, EPSC amplitude, c-Fos expression, and AMPA/NMDA ratio in the PL, while extinction training produces the same effects in the IL; none of these patterns is observed in adolescents [10]. EPSC amplitude also appeared to be non-specifically increased in adolescents compared to other age groups in both regions of the mPFC, irrespective of learning condition (i.e., fear conditioning, extinction, or control), creating a ceiling effect that may ostensibly interfere with circuit-specific activation and LTP. Interestingly, this adolescent increase in basal synaptic transmission appears to be specific to the PFC—the same increased activity is not observed in the BLA [58]. However, like the adolescent PFC, the adolescent BLA also fails to show learning-dependent synaptic potentiation following fear conditioning, an effect seen in the juvenile and adult BLA [58]. This suggests that mechanisms other than a ceiling-effect interference with LTP are contributing to adolescent suppression of learning-dependent plasticity. 

We have found that, in addition to extended extinction training, treatment with the partial NMDA-agonist D-Cycloserine (DCS) immediately following extinction training can enhance extinction retention in adolescents [11,59]. DCS-mediated improvements in extinction retention were also associated with increased pMAPK in the PFC after extinction training and testing. Further, adolescents also show improved extinction retention and increased pMAPK expression in the mPFC when they receive twice the amount of extinction training [12], suggesting that the lack of prefrontal recruitment during extinction training associated with extinction retention deficits can be overcome. This demonstrates that if an excitatory transmission is pushed above a certain threshold, normal learning-dependent plasticity can be restored in adolescence. It is possible that prefrontal learning-dependent plasticity could be modulated by developing BLA inputs that emerge during adolescence. Evidence for this idea comes from studies in adults demonstrating that direct stimulation of the BLA (or exposure to early life stress) immediately prior to a test of extinction retrieval blocks LTP in the mPFC and causes an increased return of fear [60]. The same study also reported that treatment with the NMDA receptor antagonist MK-801 recapitulated the effects of BLA stimulation/stress exposure on mPFC LTP, while DCS rescued stress-induced impairments in mPFC LTP. Together, these results suggest a model wherein a developmentally-driven increase in BLA→mPFC transmission during adolescence (discussed further in Section 5.1) may disrupt extinction-dependent LTP in the mPFC by dampening NMDA receptor responsivity in the same manner of direct BLA stimulation or previous stress exposure.

## 4. Development of Inhibitory Networks

Deficits in extinction retention typical of adolescents may also be influenced by shifting excitability and inhibition in fear-modulatory networks over development. Although inhibitory neurons in the BLA and mPFC are composed of several different subpopulations, we will focus primarily on a class of fast-spiking interneurons expressing the calcium-binding protein parvalbumin (PV). Inhibitory PV interneurons are critical for shaping network activity underlying cognition and memory [61]; these GABAergic cells target both pyramidal neurons and other inhibitory interneurons, enabling both direct inhibition as well as disinhibition of excitatory principal neurons [61]. PV interneurons also have a prominent role in generating neuronal oscillations by synchronising the firing patterns of excitatory neurons; in the PL, this process drives fear expression [62], and in the BLA, different PV-coordinated oscillation frequencies drive fear expression vs. fear extinction by changing functional connectivity between the BLA and mPFC [63]. In this section, we review developmental changes in PV interneurons and consider how these changes may impact fear processing in adolescence.

### 4.1. Prefrontal Inhibition

Levels of PV significantly increase in the mPFC during adolescence [64,65], reflecting a heightened capacity for local inhibition. As the numbers of PV neurons in the PL and IL are similar in juveniles, adolescents, and adults [66,67], this change appears to be driven by increased growth and proliferation of PV cell neurites, meaning that existing interneurons are dramatically arborising and increasing their capacity to integrate signals and regulate activity in pyramidal neurons during adolescence. At the same time, excitatory drive onto this inhibitory population (in the form of both synaptic contacts and glutamatergic transmission) effectively doubles [64,68]. While excitation of inhibitory interneurons in the rat mPFC increases throughout development, it was recently demonstrated that excitatory synapses on inhibitory interneurons in the monkey dlPFC are actually *pruned* over adolescence [69], just as they are on excitatory pyramidal neurons (as discussed in Section 2.1). Though this pattern could be a primate specialisation, it is also possible that development of inhibitory networks may follow different trajectories in later developing prefrontal areas, in which case, one might expect to see pruning of excitatory input to inhibitory neurons in the primate and rodent OFC, though to our knowledge this has not been explored.

The increase in inhibitory tone over the course of adolescence in the mPFC coincides with enhanced GABAergic control of local field potentials. Evidence suggests that relative to adulthood and late adolescence, early adolescence is associated with reduced GABAergic inhibition of glutamatergic pyramidal neurons in the mPFC [70]. This is observed as a failure in the suppression of prefrontal local field potentials in response to high-frequency ventral hippocampal stimulation (20 and 40 Hz) in early adolescence. Ventral hippocampal-mediated long-term depression only emerges in late adolescence, suggesting delayed maturation of GABAergic interneuron function. Other studies have identified signs of increased prefrontal activity in infant and juvenile animals relative to adults (although adolescents were not included) [71], suggesting a linear increase in GABAergic control of prefrontal excitability that stabilises in late adolescence. Importantly, disrupting NMDA receptor-mediated transmission during adolescence (via systemic administration of the antagonist MK-801) from P35–49, but not adulthood, rendered this early adolescent profile of PFC disinhibition long-lasting, such that it was observed well into adulthood [70]. This enduring effect in adult rats after peri-adolescent NMDA receptor disruption was reversed by increasing local GABAergic transmission in the PFC with a single local infusion of the GABAA positive allosteric modulator Indiplon. Evidence suggests that earlier disruption of NMDA signalling (via ketamine or MK-801 during the second-third postnatal week) has similar long-term effects in adulthood, reducing expression of PV, disrupting synaptic properties in interneurons, and causing disinhibition of pyramidal cells [72,73]. Taken together, these findings demonstrate (1) that GABAergic control of prefrontal excitability increases across development before reaching mature functional capacity in late adolescence, and (2) that sustained NMDA receptor transmission is critical for moderating the normal functional development of GABAergic inhibitory networks in the mPFC. 

Behaviourally, this developmental trajectory of prefrontal inhibitory networks could significantly affect how memories are acquired, stored, and retrieved at different ages. For instance, transgenic mice bred with a mutation that causes loss of PV neurons in the PFC (but not amygdala or hippocampus) show specific deficits in the extinction of cued fear, but not in its acquisition or expression [74]. Given that extinction retention is intact prior to adolescence when PV neurons are still highly immature, it appears that the mechanisms underlying fear extinction may transition from a PV-independent form in juveniles to a PV-dependent form in adolescence and adulthood, leaving adolescents in a compromised transitional period characterised by impaired extinction processing.

### 4.2. BLA Inhibition

Although the developmental trajectory of interneuron function in the amygdala has been studied less extensively than in the PFC, the available evidence suggests that PV expression in the rodent basolateral amygdala complex undergoes dynamic changes in periadolescence, and that these changes could have dramatic effects on fear regulation. Berdel and Moryś [75] found that within the magnocellular part of the basal nucleus of the amygdala, the number of PV neurons rapidly increased after P17 and peaked at P21. Density decreased between P21 and P30, and then remained stable through adulthood (P90). In contrast, PV staining in the lateral nucleus of the amygdala was not detected at all at P17 or P21 in this study; it became apparent only at P30 and remained at the same level until P90. Within the basal nucleus, the distribution of PV neurons was largely restricted to the magnocellular region at P17 and P21 and only spread to the parvicellular component at P30 (information about the number of PV neurons in the parvicellular component across development was not provided). It should be noted that using a different antibody than Berdel and Moryś [75], our group detected PV-immunoreactive neurons in the lateral nucleus of juveniles (P24), adolescents (P35–36), and adults (P70), and did not observe changes in the number of labelled cells across development in this region [66]. Within the entirety of the BLA complex (lateral+basal nuclei), we did observe a trend (*p* = 0.052) towards a loss of PV neurons between juveniles and adolescents that appeared to be driven largely by changes in the basal nucleus. Although we did not distinguish between the magnocellular and parvicellular components of the basal nucleus in our study, our results may partially replicate the reduction in PV staining from juvenility to adolescence in the magnocellular basal nucleus found by Berdel and Moryś. It is important to note that the loss of PV staining could reflect a loss of neurons and/or a reduction in PV protein expression. In terms of implications for fear regulation, the anterior magnocellular part of the basal nucleus is connected more prominently to the PL, whereas the IL interacts more with the posterior parvicellular component [49]; differential development of inhibitory networks in these separate regions could therefore create a temporary imbalance between the strength of fear and extinction pathways across development, as the PL-amygdala fear pathway may mature earlier than the IL-amygdala extinction pathway. 

Functionally, recent evidence demonstrates that PV interneurons in the BLA have a critical role in modulating relapse of extinguished fear. PV neurons in the BLA are strategically located to modulate PFC activity via BLA to PFC projections. Davis et al. [63] silenced PV interneurons in the BLA using a selective chemogenetic approach coupled with activity-based neuronal-ensemble labelling and electrophysiology. This approach allowed the authors to tag neurons that were active during fear conditioning and examine the effect of PV-silencing specifically on the activity of these identified “fear” neurons. When PV neurons were silenced, BLA fear neurons were disinhibited; as a result, there was also increased activation of neurons in the PL. This BLA-PL fear circuit appears to be important for regulating freezing after extinction, as PL activity associated with BLA dis-inhibition correlated with freezing after extinction (i.e., at an extinction retention test for contextual fear learning). An opposite effect was found in IL, where activity was inhibited following silencing of BLA PV neurons. These findings suggest that impairments in extinction retention could be driven by poor (or immature) functioning of BLA PV neurons, which, in turn, results in disinhibited activation of BLA fear neurons, robust activation of the BLA-PL “fear network”, and suppression of the BLA-IL “extinction network”. Although this hypothesis (i.e., hypofunctioning of BLA PV neurons during development) has not explicitly been tested, there are indications that inhibitory transmission in the BLA undergoes dramatic changes in adolescence. The mechanisms underlying GABAergic transmission onto BLA pyramidal neurons (i.e., GABA receptor subunit expression, rise/decay time of GABA currents, etc.) are mature in the rat by P28 [76], just at the transition between the juvenile period and adolescence. However, changes in spontaneous inhibitory transmission continue throughout adolescence, suggesting a protracted developmental trajectory for amygdala interneurons. Within the basal nucleus, both spontaneous inhibitory postsynaptic current (sIPSC) frequency and the sIPSC:sEPSC frequency ratio increase from P10 to P30, and then gradually decline into adulthood [77]. In contrast, sEPSC frequency increases sharply between P10 and P15, then remains relatively stable into adulthood. A different pattern is observed in the lateral amygdala; in this nucleus, both sIPSC frequency and the sIPSC:sEPSC frequency ratio increase linearly from infancy through adulthood [78]. As the basal nucleus is the target of prefrontal innervation, it seems likely that some of this periadolescent fine-tuning may be influenced by changing connectivity across development. To explore this possibility further and examine its functional implications, we must consider the postnatal maturation of the robust reciprocal projections between the prefrontal cortex and amygdala.

## 5. Connectivity

**Figure 2:**
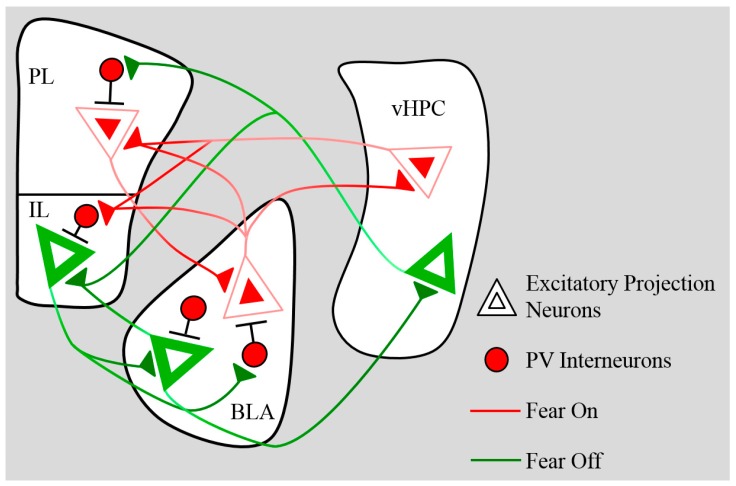
Pathways for Fear and Extinction. Projection neurons in the basolateral amygdala (BLA) and ventral hippocampal (vHPC) target both excitatory neurons and inhibitory interneurons in the medial prefrontal cortex (mPFC). The mPFC targets excitatory and inhibitory cells in the BLA, although innervation of BLA parvalbumin (PV) interneurons comes more strongly from the infralimbic (IL) than the prelimbic (PL). Red pathways represent proposed mechanisms for fear expression, whereas green pathways would promote extinction.

The PFC and the amygdala are strongly connected via robust reciprocal projections. These projections undergo substantial anatomical and functional changes over development that have the potential to dramatically impact the storage and retrieval of fear-related memories. Pyramidal BLA-mPFC projection neurons target both excitatory principal neurons and inhibitory interneurons in the opposite structure, creating complex postsynaptic events that change over the course of development. A summary of pathways established in adult rodents is shown in Figure 2. In this section, we review the anatomical and functional maturation of projections between the mPFC and the BLA, and discuss regulation of this pathway by the ventral hippocampus.

### 5.1. BLA→mPFC

#### 5.1.1. Anatomical

Infusions of retrograde tracers into the mPFC in developing mice reveal an increase in the density of neurons projecting from the BLA to the PL from juvenility through early adolescence (i.e., from P23 to P30) and a subsequent decrease (at P45) in late adolescence [36]. In the same study, no changes in connectivity were identified between the BLA and IL (*p* = 0.056), albeit this study had a smaller sample size, suggesting further investigation with larger sample sizes (i.e., >4 per group) might reveal developmental changes in BLA to IL projections. Complementary experiments using anterograde tracers in the BLA show that fibres from the amygdala develop a progressively clear bilaminar pattern with age; fibre density increases in layers II and V of the IL and PL from birth through late adolescence, levelling off in adulthood [79]. This suggests that even as the number of BLA neurons projecting to the mPFC is pruned, the remaining connections continue to mature and strengthen. Cunningham et al. [79] also demonstrated that the percentage of contacts between BLA fibres and the spines, dendrites, and axons of PFC neurons increased linearly with age, while the percentage of fibres making no contacts showed an equal and opposite decrease, providing further evidence that maturing BLA→PFC projections increase in functional capacity with age. It is interesting to consider that while Cunningham et al. [79] reported a linear increase in BLA→mPFC axospinous synapses (excitatory contacts between BLA axon terminals and PFC dendritic spines) over development, synapses, and spines in the PFC overall undergo massive pruning in adolescence, as discussed in Section 2.1. This may suggest that synaptic pruning occurs only in select pathways, presenting opportunities for newly forming patterns of innervation to emerge. As excitatory afferents from distal forebrain sites like the amygdala and hippocampus are slow to arrive in the PFC, one might predict that much of the excitatory innervation of the PFC prior to adolescence is derived from thalamocortical and local corticocortical connectivity, and that these may be the synaptic connections that are more vulnerable to pruning during adolescence.

#### 5.1.2. Functional

Activation of BLA→prefrontal projections induces long-term potentiation (LTP) in the mPFC. This effect is functional by P30 but still maturing, evidenced by larger increases in mPFC local field potentials following BLA stimulation in adults compared to adolescents [80]. This effect was not dependent on GABA, indicating that BLA facilitation of prefrontal LTP is driven by innervation of pyramidal neurons in the mPFC and does not require recruitment of interneurons. It should be noted that these findings do not imply that BLA innervation of prefrontal GABAergic neurons is not biologically or behaviourally relevant; it is entirely possible that discrete activation of BLA subpopulations during complex learning events could selectively stimulate prefrontal GABAergic transmission. For instance, unlike BLA-evoked prefrontal LTP, ventral hippocampal (vHPC) stimulation induces long-term depression (LTD) in the mPFC; this effect emerges later in development (after P55), and is dependent on GABAergic transmission [80]. If inputs from the vHPC and the BLA send axon collaterals to the same PV interneuron, stimulation of that interneuron could drive feedback inhibition onto both inputs, ultimately synchronising the firing pattern of PFC-projecting BLA and vHPFC neurons. Indeed, it has been shown that 1) hippocampal and amygdala afferents converge on neurons in the IL and ventral PL, 2) excitatory responses of these neurons are significantly amplified by simultaneous vHPC+BLA stimulation, and 3) staggered stimulation of BLA and vHPC (separated by 20–40 ms) has an inhibitory effect on the postsynaptic neuron [81]. This suggests that synchronisation of BLA and vHPC firing in the mPFC by PV-mediated feedback inhibition could significantly drive activity and plasticity necessary for the complex integration of cues and context. The fact that desynchronised activity can have inhibitory effects on postsynaptic neurons may also explain the lack of learning-dependent plasticity observed in adolescents (see Section 3). As the PFC is receiving growing input from different brain regions while PV interneurons are still underdeveloped, disorganised excitatory input (while increasing basal synaptic transmission) could actually decrease the probability of action potentials in PFC neurons and blunt learning-dependent changes. 

### 5.2. mPFC→BLA

#### 5.2.1. Anatomical

Neurons in the mPFC that project to the BLA are predominately located in the same layers that receive innervation from the amygdala; BLA-projecting neurons are present in superficial layers II/III and deep layer V across the mPFC [63,82,83,84,85,86]. These neurons receive excitatory inputs from the ventral hippocampus and are subject to inhibitory regulation through local PV interneurons [87]. The number of neurons projecting from the PL and IL appears to be relatively balanced in adults, as infusions of the retrograde tracer CTB in the BLA typically labels similar numbers of neurons in the PL and IL [63]. However, these subregions differ in terms of the types of cells in the BLA that they contact. While projections from the PL and the IL both preferentially innervate the basal nucleus of the BLA, the IL sends stronger inputs to the parvicellular aspects, while the PL more selectively innervates the magnocellular population [49]. In addition, the IL sends more projections to PV BLA interneurons than the PL [63]; given that PV interneurons in the parvicellular division are late to develop (see Section 4.2), this suggests that IL-domination of PV inhibition in the amygdala may not be functionally mature until relatively late in adolescence, and that earlier in development, the balance of control may be tipped more towards the PL.

Unlike bottom-up BLA→mPFC connectivity, top-down projections from the mPFC to the BLA undergo pruning of both fibres and the total number of projection neurons from adolescence through adulthood. Retrograde tracing showed that the number of IL neurons that project to the BLA decreases linearly from juvenility (P25) through adulthood while PL→BLA neurons show a delayed pruning pattern, remaining stable from P25 to late adolescence (P45) before sharply decreasing in number to reach adult levels at P90 [88]. The same study also used anterograde tracing to further examine pathway development, and found that fibres in the BLA originating from the mPFC (PL+IL) maintain a similar density from P25–P45 and are then pruned from late adolescence through adulthood. Another study that examined a broader window of development (six time points from P10–P80) revealed massive mPFC→BLA fibre proliferation between P10 and P30, and confirmed a modest decrease later in adolescence between P45 and adulthood [77]. These results suggest an overall increase in mPFC innervation of the BLA with age, with a discrete period of pruning and reorganisation occurring in late adolescence.

#### 5.2.2. Functional

Stimulation of mPFC afferents in the amygdala coupled with single cell recordings of principal neurons of the BLA (specifically, the basal nucleus) reveal a strengthening of glutamatergic mPFC-amygdala synapses in early development that plateaus by P30 and remains stable through adulthood [77]. However, the same study found that disynaptic inhibitory transmission (i.e., mPFC pyramidal neuron → BLA interneuron → BLA pyramidal neuron) massively increased in amplitude between P21 and P30, and subsided again at P45 and P60. This led to a temporary surge in the IPSC:EPSC amplitude ratio in early adolescence that was more than double the values observed in adulthood, indicating that the mPFC has the capacity to drive substantial GABAergic transmission in the amygdala for a transient period at the onset of adolescence. While the mechanisms for this robust inhibitory potential are as yet unclear, it is possible that the peak in mPFC innervation of the BLA at P30 reflects increased prefrontal targeting of amygdala inhibitory interneurons, and that these synapses are preferentially eliminated between adolescence and adulthood; this could account for the observed reduction in fibre density during this period. Importantly, the findings of Arruda-Carvalho et al. [77] using in vitro stimulation stand in stark contrast to the effects observed using an in vivo stimulation approach. Selleck et al. [89] analysed local field potentials and single-unit recordings in the BLA following electrical stimulation of the mPFC in anaesthetised rats [89]; the findings of this study suggest that prefrontal modulation of amygdala activity is significantly blunted in adolescents compared to adults, contrary to the increased inhibitory control reported by Arruda-Carvalho et al. [77]. In this approach, mPFC stimulation at 10–20 Hz induced local field potential facilitation in the basal nucleus of the BLA of adults, but was ineffective in adolescents (P39). Further analyses showed that in both adolescents and adults, the majority of BLA neurons (~65%) showed an inhibitory response to mPFC stimulation; however, BLA inhibition evoked by stimulation of both the PL and the IL was weaker in adolescents than adults. This pattern of increasing prefrontal inhibitory control of the amygdala from adolescence to adulthood has also been demonstrated in humans [90], and nicely fits the model of compromised emotional regulation in adolescents. Unfortunately, as younger age groups were not included in the in vivo analyses, it is unclear whether the observed impairment in prefrontal regulation of amygdala activity is specific to adolescence or simply a reflection of immature connectivity that would also be observed in juveniles. 

Aside from different methodological approaches, what might explain the contradictory findings of Selleck et al. [89] and Arruda-Carvalho et al. [77] concerning mPFC-evoked inhibitory drive in the adolescent BLA? For one, stimulation of the entire mPFC (Selleck et al.) could have more complex downstream effects than discretely driving mPFC terminals within the BLA (Arruda-Carvalho et al.). The former approach would stimulate excitatory and inhibitory cell bodies, dendrites, and afferents within the mPFC and would drive mPFC-BLA network activity in both direct and indirect (i.e., mediated by thalamic or hippocampal relay) pathways. This in vivo approach has obvious advantages in that it reveals how the mPFC coordinates amygdala activity in an intact, biologically-relevant system with information being integrated from multiple networks. However, by excluding all additional activity and honing in on a precise synaptic event, the in vitro approach may reveal more about the precise anatomical and functional development of mPFC-BLA pathways. In any case, when taken together, the results of these two studies show that the mPFC does not effectively inhibit amygdala activity in adolescents under normal conditions, but indicate that it nonetheless has the *capacity* to induce massive inhibition at this developmental stage; this suggests that mPFC→BLA inhibitory transmission is experiencing interference upstream of the BLA during adolescence. Disruption of mPFC-BLA functional connectivity is likely a major contributor to adolescent impairments in extinction retention and future work aimed at facilitating connectivity between these structures may lead to innovative treatment approaches for adolescent-onset anxiety.

### 5.3. Ventral Hippocampus→mPFC

The BLA-mPFC network obviously does not exist in isolation; as referenced in previous sections, the ventral hippocampus (vHPC) is a critical third node in this fear-regulatory forebrain network. Like the mPFC and the BLA, the vHPC undergoes substantial changes in connectivity across development that critically impact fear- and extinction-related processes. 

As previously discussed (Section 4.1 and Section 5.1.2), vHPC and BLA inputs into the PFC functionally mature over different developmental time windows (P30 for the BLA and ~P55 for the vHPC) and recruit distinct forms of plasticity in the PFC (LTP for the BLA vs. LTD for the vHPC). The vHPC is a powerful regulator of BLA-mPFC functional connectivity; in adults, stimulation of vHPC inputs with high frequency exerts inhibitory control over BLA drive to the mPFC [91]. Given the late emergence of vHPC-mPFC functional connectivity, this suggests that in early adolescence, the BLA projections to the mPFC are not yet dampened by vHPC regulation; this may contribute to the mPFC being over-responsive to BLA inputs at this age. 

In the adult mouse, the vHPC innervates pyramidal neurons situated in both superficial layers II/III and deeper layer V of the IL, but only layer V of the PL [92]. As previously discussed, BLA and vHPC inputs predominantly converge in the IL and the ventral aspects PL. Projections between the vHPC and the PL surge between P23 and P30, peaking in early adolescence before pruning in late adolescence and adulthood [36]. This means that maturation of vHPC→mPFC anatomical connectivity precedes the development of functional connectivity in the form of vHPC-evoked prefrontal LTD and inhibitory control of BLA→mPFC inputs. Like the BLA, the vHPC targets both pyramidal neurons and PV interneurons in the mPFC [87] (see Figure 2). It could be speculated that these early-emerging projections from the vHPC primarily target pyramidal neurons in the PFC, with innervation of interneurons developing later. A simpler (not mutually exclusive) explanation is that prefrontal PV interneurons receive vHPC input in early adolescence but are not yet mature enough to mediate inhibitory transmission at levels sufficient to induce effective vHPC-evoked LTD and synaptic dampening. 

vHPC-mediated control of the IL appears to be composed of two functionally disparate pathways: a pro-extinction Brain-Derived Neurotrophic Factor (BDNF)-dependent excitatory pathway and a pro-fear inhibitory pathway. In general, the inhibitory pathway appears to be the default; driving activity in vHPC→IL projections induces PV-mediated feedforward inhibition onto IL pyramidal neurons, and both broad activation of the vHPC and selective activation of vHPC→IL projections results in increased recovery of fear [87]. In contrast, extinction training induces BDNF production in the vHPC, which has been shown to increase the firing rates of IL (but not PL) neurons and facilitate acquisition and retention of extinction [93,94]. Notably, Rosas-Vidal et al. [93] found that while most of the neurons recorded in the PL showed no change in response to vHPC BDNF, approximately 30% were inhibited (compared to the excitatory effect on IL neurons). However, recordings appear to have been taken from both deep and superficial layers of the PL, with a slight bias towards superficial layers II/III; if recordings were restricted to layer V (which receives the majority of vHPC inputs), it is possible that a more consistent inhibitory response would have been observed in the PL. Inactivating the vHPC prior to extinction has also been shown to impair extinction acquisition and retention [95]. This suggests that while inhibition may be the dominant force mediating vHPC-IL interactions, during extinction learning hippocampal BDNF selectively facilitates activity in an excitatory vHPC-IL pathway that is necessary for fear suppression. vHPC-mediated control of the PL is also prominently regulated by BDNF. Inhibiting BDNF activity in the vHPC during adolescence both impairs extinction learning and causes diminished vHPC innervation of the PL (but not the IL) in adulthood [96]. Taken together, these findings demonstrate that the vHPC can differentially modulate activity in the mPFC to either promote fear expression or facilitate extinction. Delayed functional development of the pro-extinction BDNF-dependent pathways relative to the vHPC→IL pro-fear inhibitory pathway may be a contributing factor driving increased fear relapse in adolescence, but this remains a question for future research.

## 6. Disruption by Chronic Stress

Considering the massive changes in brain structure and function discussed above, it is no great surprise that adolescence represents a significant window of vulnerability that renders the developing animal particularly sensitive to environmental insults. These potential insults are wide-ranging and include poor diet, drugs of abuse, and many other damaging influences, but for the purposes of this discussion we will focus on the effects of chronic stress. Here we focus on how exposure to external stressors during adolescence disrupts the normative developmental trajectory and results in persistent changes to behaviour such as fear regulation. However, neurodevelopment and emotional behaviour are affected by adverse experiences throughout the lifespan, including prior to conception [97], prenatal [98] and early postnatal periods [99], and so we direct the reader to excellent reviews on those topics. 

In terms of effects on fear extinction in adolescence, chronic stressor exposure by restraint or social instability in adolescence impairs the acquisition or retention of extinction memories when tested in adolescence relative to non-stressed controls [100,101,102]. Adolescence appears to be a particularly stress-sensitive developmental period in terms of fear regulation because animals are more susceptible to extinction deficits when stress occurs during adolescence compared to when it occurs in the juvenile period [103] or adulthood [102,104]. Further, such deficits induced by adolescent stress are long-lasting, persisting into adulthood [104]. The consequences of adolescent stress on fear extinction are important clinically when considering strategies for chronically stressed youth presenting for treatment of anxiety disorders. This is because chronic exposure to the stress hormone corticosterone in adolescence reduces the benefit of two approaches that augment extinction retention in adolescent rats, namely extra extinction training [103,104] and pharmacological augmentation by DCS [103]. Such results suggest that a history of chronic stress could further reduce the efficacy of anxiety treatments in adolescents.

The enduring effects of adolescent stress may be caused by maladaptive developmental trajectories of subcortical and cortical emotion regulation systems. In support of this claim, there is evidence that human adolescents with traumatic stress exposure (in late childhood or adolescence) have weaker PFC-amygdala connectivity (reviewed by [2]). Further, in rodents, chronic exogenous corticosterone exposure in adolescence diminishes neuronal activity in the mPFC via a down-regulation of glutamatergic receptors (e.g., NMDA receptors [105]), which are essential for extinction consolidation, and induces structural changes by simplifying hippocampal dendritic structure and altering neuronal spine density in the IL, OFC, hippocampus, and amygdala [106]. Specifically, adolescent corticosterone exposure (for 20 days in mice aged between 5 and 7 weeks old) reduces spine density on pyramidal neurons in the IL (in deep layers), OFC, and hippocampus but has the opposite effect in the amygdala [106]. Although some of these changes were transient and recovered once stressor exposure ended, others such as spine reductions in the OFC and dendritic arborisation in the CA1 were long-term effects that persisted after the stressor had ended. 

Earlier onset of chronic stress may induce more persistent, and opposite, effects on BLA spine density. For example, one study reported both immediate and long-term reductions in spine density of BLA pyramidal neurons following restraint stress during juvenility and early adolescence (i.e., 2 h daily restraint from P21 to P35; [107]). The reduction in spinogenesis in the BLA from chronic stress in the juvenile-adolescent period contrasts with reports of increased spinogenesis and dendritic hypertrophy resulting from stress later in adolescent development (described above [106]) or adulthood [108]. Further, chronic restraint stressor exposure in adolescence can induce changes in dendritic morphology in the dorsal mPFC that are layer specific [107], suggesting that synaptic communication of specific afferents (e.g., from the BLA) could be impaired. 

The transition from juvenility to adolescence is also a time of maturation of prefrontal and amygdala perineuronal net (PNN) maturation around PV inhibitory interneurons [66] and so it is not surprising that chronic stress occurring during this time reduces inhibitory neuron expression in the PL [109] and alters PNN expression in the PFC (in the OFC) [110]. The stress-induced loss of PV interneurons may reduce local inhibition of pyramidal neurons in the PL and shift this region towards a more “pro-fear” state. Other work links adolescent stress-induced deficits to impaired function of the IL. For instance, chronic unpredictable and chronic restraint stress in adolescence impair fear extinction in adulthood through reductions in basal levels of BDNF and activation of its principal receptor (i.e., pTrkB) and downstream pMAPK signalling in the IL [111]. These cellular mechanisms in the PL and IL may therefore lead to increased fear despite extended extinction training in stress-exposed male adolescent animals [104]. Taken together, the findings discussed in this section illustrate that chronic stress in adolescence alters pyramidal cell and interneuron structure and function, developmental trajectories of PNN maturation, and suggest potential mechanisms by which adolescent stress might impair extinction. Future studies could further investigate the functional consequences of stress in adolescence, such as whether there is reduced synaptic plasticity in reciprocal connections between the BLA and PFC during extinction, and whether the neural and behavioural effects of adolescent stress can be prevented or rescued. 

## 7. Conclusions

**Figure 3:**
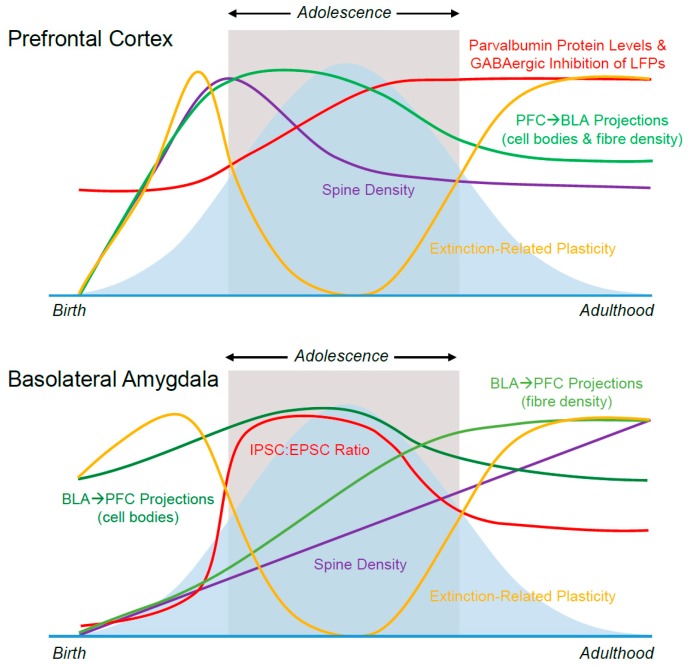
Summary of Neuroanatomical Changes Across Development. Blue bell curve represents fear relapse following extinction. Key: PFC, prefrontal cortex; BLA, basolateral amygdala; LFPs, local field potentials; I/EPSC, inhibitory/excitatory postsynaptic current.

In this review, we summarise developmental changes in neuroplasticity, inhibition, and connectivity that occur within and between the mPFC and amygdala in adolescent animals, see Figure 3. We suggest ways in which these changes may influence the characteristic deficits in extinction retention displayed by adolescents, and consider how these delicately-modulated neural processes may be influenced by exposure to stress. It should be noted that the developmental differences discussed here are by no means comprehensive; many other variables, including (but not limited to) dopaminergic infiltration of the PFC, exposure to sex hormones, and changing levels of neurotrophins/neurotrophin receptors likely have dramatic impacts on fear learning and memory in adolescence. Creating a comprehensive roadmap of the complex and dynamic mechanisms underlying emotional processing in the adolescent brain continues to be a massive undertaking; however, increasing interest in the field means that the body of knowledge in this area is swiftly proliferating. Thanks to valuable research contributions like those described above, we are steadily advancing towards the development of targeted treatment approaches designed to meet the storm and stress of adolescence head-on.

## Abbreviations

BDNF	Brain-Derived Neurotrophic Factor
CS	Conditioned stimulus
(m)PFC	(medial) Prefrontal Cortex
IL	Infralimbic
PL	Prelimbic
OFC	Orbitofrontal Cortex
dlPFC	Dorsolateral Prefrontal Cortex
BLA	Basolateral Nucleus of the Amygdala
vHPC	Ventral Hippocampus
PV	Parvalbumin
LTP	Long-Term Potentiation
LTD	Long-Term Depression
(s)EPSC	(spontaneous) Excitatory Postsynaptic Current
(s)IPSC	(spontaneous) Inhibitory Postsynaptic Current
AMPA	Alpha-Amino-3-Hydroxy-5-Methyl-4-Isoxazolepropionic Acid
NMDA	N-Methyl-D-aspartatic acid
DCS	D-Cycloserine
GABA	Gamma-Aminobutyric Acid
P	Postnatal Day
PNN	Perineuronal Nets
UCS	Unconditioned stimulus

## Appendix A. Guide to Rodent Development in (Approximate) Postnatal Days

<7—Perinatal

P7–21—Infancy

P22–P27—Juvenility

P28–P35—Early Adolescence

P36–P41—Mid-Adolescence

P42–P55—Late Adolescence

>P60—Adulthood

*See [30,112]. Values are based on rat development, but a similar trajectory is observed in mice.
